# Dissemination of CTX-M-Producing *Escherichia coli* in Freshwater Fishes From a French Watershed (Burgundy)

**DOI:** 10.3389/fmicb.2018.03239

**Published:** 2019-01-08

**Authors:** Loic Bollache, Emeline Bardet, Géraldine Depret, Sébastien Motreuil, Catherine Neuwirth, Jérome Moreau, Alain Hartmann

**Affiliations:** ^1^UMR CNRS 6249, Laboratoire Chrono-Environnement, Besançon, France; ^2^Agroécologie, AgroSup Dijon, INRA, Université de Bourgogne, Université de Bourgogne Franche-Comté, Dijon, France; ^3^UMR CNRS 6282 Biogéosciences, Laboratoire Biogéosciences, Dijon, France; ^4^Bacteriology Department, University Hospital, Université de Bourgogne Franche-Comté, Dijon, France

**Keywords:** freshwater, ESBL producing *Escherichia coli*, *bla*_CTX–M_, class 1 integron-integrase, antibiotic resistance, fish, MLST *E. coli*

## Abstract

The burden of extended-spectrum β-lactamases producing *Escherichia coli* (ESBL-*Ec*), has increased over several decades. Freshwater ecosystems are suspected to play an important ecological and evolutionary role in driving the dissemination of antimicrobial resistance. The aim of our study was to decipher the occurrence of ESBL-*Ec* in a small watershed (Ouche river, Burgundy, France), targeting environmental matrices and fishes. Among cefotaxime resistant *E. coli* (ctxR *Ec*) isolates, we detected and characterized 36 ESBL-*Ec* from water, biofilm and fish guts. ctxR *Ec* and ESBL-*Ec* were found in samples from sites near the first small town, located downstream from the watershed which was studied. Treatment of urban wastewater by waste water treatment plants (WWTP), might therefore be a major potential source of ctxR *Ec* and thus of ESBL-*Ec*. Prevalence of total *E. coli* and ctxR *Ec* in fish guts ranged between 0 to 92% and 0 to 85%; respectively, depending on the sampling site and the fish species. The diet of fish (predator or omnivore) seems to strongly influence the prevalence of total *E. coli* and ESBL-*Ec*. Extended spectrum beta-lactamases produced by the isolates from this study belonged to the CTX-M family (CTX-M group 1 and 9). Moreover, some environmental ESBL-*Ec* proved to share genotypic features (MLST types) with isolates which originated from 8 WWTP effluents discharged in the Ouche river and with the sequence type ST131, which is widely described in clinical isolates. Ninety-seven % (97%) of ESBL-*Ec* from the study harbored additional antibiotic resistances and can thus be considered as multi drug resistant (MDR) bacteria. Finally, 53% of the ESBL-*Ec* strains harbored class 1 integron-integrase (*intl1*). These results are discussed with the perspective of defining indicators of antibiotic resistance contamination in freshwater ecosystems.

## Introduction

Dissemination of extended-spectrum β-lactamases (ESBLs) among *Enterobacteriaceae* (especially by *Escherichia coli)* has dramatically increased among human populations (in community and hospital settings) over several decades and is recognized worldwide as a major public health concern (Woerther et al., 2010). With increasing numbers of ESBL-producing bacteria in healthcare-associated and community-acquired infections, ESBL-producers have become one of the most important groups of multi-drug-resistant (MDR) bacteria involved in healthcare-associated infections caused by *E. coli* or other enterobacteria (Bonnet, 2004).

First described in 1989, the CTX-M type ESBL is nowadays the most prevalent clinical isolate worldwide (Livermore and Hawkey, 2005) but also the most prevalent in human and animal feces (Guenther et al., 2011), in sewage sludge (Reinthaler et al., 2010), in soils (Hartmann et al., 2012) and in aquatic environments (Zurfluh et al., 2013; Blaak et al., 2014; Maravic et al., 2015). Effluents discharged into aquatic ecosystems, by waste water treatment plants (WWTP) treating urban and hospital wastewater, have been clearly identified as a source of extended-spectrum β-lactamases producing *Escherichia coli* (ESBL-*Ec)* that are disseminated in aquatic environments (Brechet et al., 2014; Blaak et al., 2015; Franz et al., 2015).

Whereas mechanisms conferring resistance to many antibiotic families in bacteria are well understood (Tenover, 2006; Stokes and Gillings, 2011), the processes facilitating persistence and dissemination of ESBL-producing bacteria in the environment have been less intensively studied.

Among ecological and environmental factors contributing to the environmental spread of antimicrobial resistant bacteria, the epidemiological role of wild fauna as reservoirs or transporters of resistant bacteria, has been repeatedly emphasized. Thus, ESBL-*Ec* strains have been reported in many wild species, particularly in wild bird populations living in areas inhabited by people (Bonnedahl et al., 2009, 2010; Literak et al., 2010; Poirel et al., 2012; Vittecoq et al., 2016; Mohsin et al., 2017; Carter et al., 2018) and mammals (Costa et al., 2006; Literak et al., 2009; Goncalves et al., 2012). Therefore, the role of the environment and especially aquatic ecosystems, has to be assessed with regards to the emergence of resistant bacteria like ESBL-*Ec* (Taylor et al., 2011; Marti et al., 2014). Marine and freshwater ecosystems are known to receive numerous chemical and biological contaminants, among those antibiotic residues and antibiotic resistant bacteria are released in aquatic ecosystems through discharge from livestock and WWTP. However, the spread of ESBL-*Ec* into freshwater ecosystems and the role of fishes in this process have received little attention (Tacao et al., 2012; Pereira et al., 2013). Indeed, fishes are mobile, have different feeding habits and use various microhabitats in the river (Popova and Sytina, 1977; Hyslop, 1982; Weatherley, 1987; Garner, 1996), thus freshwater fishes might also disseminate ESBL-*Ec* through their movements and might serve as integrators of a freshwater ecosystems bacterial contamination. Indeed, recently several authors have documented the contamination of wild fish in marine environments (Brahmi et al., 2018) and in lakes (Abgottspon et al., 2014; Zurfluh et al., 2015; Moremi et al., 2016) as well as in farmed fish (Jiang et al., 2012; Hon et al., 2016), stressing that fishes could participate in the spread of multidrug resistant enterobacteria. However, such data is missing for fishes from rivers and streams.

Finally, freshwater might constitute an efficient vector for the spread of antibiotic resistance through various ways: i.e., irrigation of cultures or drinking water for livestock animals. Thus, freshwater ecosystems are suspected to play an important ecological and evolutionary role in driving the emergence, persistence and dissemination of antimicrobial resistance (Taylor et al., 2011), and to become a reservoir potentially leading to human contamination through water, food consumption or recreational activities (Leverstein-van Hall et al., 2011; Schijyen et al., 2015). In this context, it is important to determine how these bacteria have spread to freshwater ecosystems and their associated fauna as well as to understand the role of human activities in the occurrence of contamination. Concerning freshwater fauna, this study targets fishes occurring naturally in river ecosystems.

The objectives of the study were to evaluate (i) the occurrence of ESBL-*Ec* strains in freshwater fishes and their natural environment (water and biofilm), collected at several sites of the Ouche watershed (Burgundy, France), (ii) the diversity of ESBL-*Ec* from fishes and their environment (MLST typing), their antibiotic susceptibility and the prevalence of *bla* genes as well as the occurrence of class 1 integrons, in the isolates. The genetic diversity of environmental ESBL-*Ec* strains was compared with that observed in a collection of ESBL-*Ec* strains isolated from effluents of eight WWTP, discharging into the Ouche watershed.

## Materials and Methods

### Sites Studied Along the Ouche River and Sampling Methods

**FIGURE 1:**
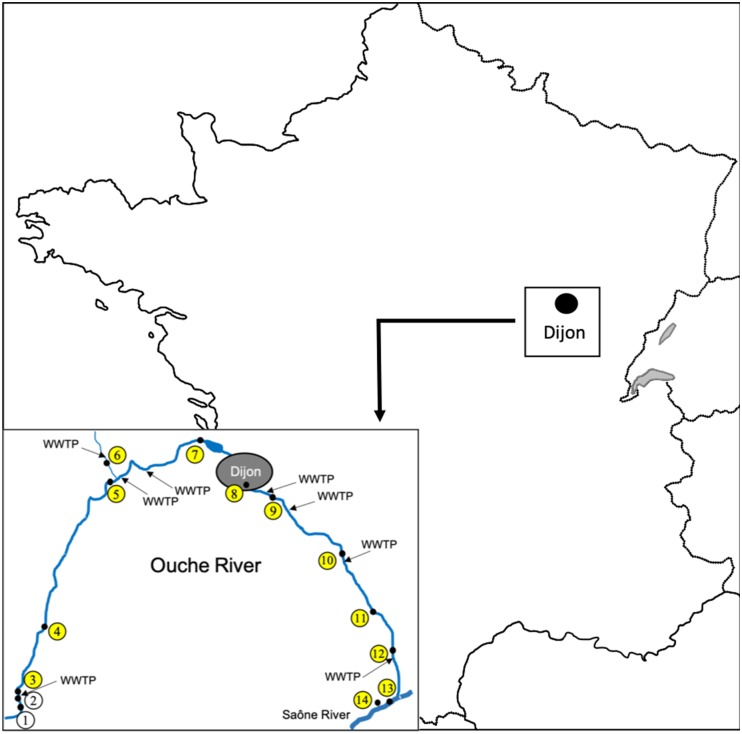
Location and map of Ouche river watershed, the position of the sampling sites is indicated by their numbers within open circles as described in Table 1, yellow colored circles indicate positive sites for the presence of ctxR *Ec.* Locations of eight WWTP discharging into the Ouche river are indicated by arrows. The bar represents a 5 km distance.

In spring 2012, 14 sampling sites were defined along the river Ouche (Burgundy, Eastern France), from the spring to the Saône confluence (Figure 1, site 1: Lusigny-sur-Ouche 47°05′25.2″N 4°40′22.0″E, site 2: Bligny-sur-Ouche, site 3: Bligny-sur-Ouche 47°06′34.6″N 4°39′58.2″E, site 4: Pont d’Ouche 47°10′02.1″N 4°42′02.7″E, site 5: Pont de Pany 47°17′49.9″N 4°48′47.5″E, site 6: Mâlain 47°19′18.1″N 4°48′22.4″E, site 7: Plombières 47°19′40.8″N 4°59′16.8″E, site 8: Dijon 47°17′41.1″N 5°02′50.0″E, site 9: Dijon 47° 17′ 11.761″N, 5° 04′ 59.883″E, site 10: Varanges 47°13′59.4″N 5°11′58.2″E, site 11: Tart l’Abbaye 47°10′55.1″N 5°15′08.2″E, site 12: Trouhans 47°08′54.8″N 5°16′38.8″E, site 13: Saint-Jean-de-Losne (Saône river) 47°06′03.4″N 5°15′58.9″E, site 14: Saint-Jean-de-Losne (harbor) 47°06′08.2″N 5°15′35.0″E). For all sites, 500 mL of water were collected (0.5 m from the shore and between 20 to 50 cm depth). One to 5 g of epilithon or biofilm were collected directly from the stone surface and from aquatic plants, using a sterile single use blade to prevent cross contaminations between sites. Fishes were sampled (one campaign) only from the sites eight and nine using battery-powered portable electrofishing gear (Dream Électronique Society, France). All fishes were maintained in aerated 40 L plastic tanks until their arrival at the laboratory. Within 4 h after collection, fishes were identified to the species level according to Keith and Allardi (2001) and measured (fork length). A total of 14 water samples, 16 biofilm samples and 127 fish samples were collected and used for further microbiological analyses as described below. The locations of the eight WWTP discharging treated effluents into the Ouche river are available in Figure 1.

### Detection, Enumeration and Isolation of Total *E. coli* and Cefotaxim Resistant *E. coli* (ctxR *Ec*) From Water, Biofilm Samples and From Fish Guts

Water sample processing: Four hundred milliliters of water samples were filtered on HAWP cellulose ester filters (porosity 0.45 μm) (Merck Millipore, Germany). Filters were then washed into 5 mL of buffered peptone water (BPW) (AES Chemunex, bioMérieux France) and mixed vigorously with a vortex to resuspend bacteria.

Biofilm sample processing: Epilithon and biofilm samples were resuspended in 5 mL of BPW and homogenized by vigorous shaking.

Fish sample processing: Fishes were anesthetized and gutted using aseptic techniques immediately upon arrival at the laboratory. The fish guts were then rinsed with sterile BPW. Guts were ground and homogenized in 1 mL of BPW by adding 0.1 g of 106 μm diameter glass beads (Sigma-Aldrich, United States) and vortexed for 2 min. The homogenized gut suspension was diluted with 9 mL of BPW.

Then, 100 μL of BPW suspensions which originated from either water, biofilms and homogenized gut samples were spread onto Tryptone Bile X-glucuronide agar plates (AES Chemunex, bioMérieux France), supplemented or not with 4 mg/L of cefotaxime (Sigma-Aldrich, United States), in order to detect and enumerate ctxR *Ec* and the total *E. coli*, respectively. Plates were incubated at 37°C for 18 h. To maximize the sensitivity of the detection of ctxR *Ec*, a non-selective enrichment step was added: i.e., the remaining BPW suspensions were incubated at 37°C for 24 h and then 100 μl of this enrichment were plated as described above. After incubation, blue colonies (glucuronidase positive colonies) were counted. For enriched samples, results were expressed as positive (presence of blue colonies) or negative (absence of blue colonies).

For each (enriched or not) plate, 3 ctxR isolates were randomly picked up and purified twice on TBX, supplemented with cefotaxime (4 mg/L) plates. Isolates were stored at -80°C in a Luria Bertani medium (AES Chemunex, bioMérieux France) supplemented with 25% glycerol.

### Phenotypic Characterization and Antibiotic Resistance Testing of ctxR *Ec*

Cefotaxim resistant isolates were confirmed to belong to the *E. coli* species, by testing their capacity to ferment lactose on Drigalski agar media (Conda, Spain) and by testing on an API 20E test kit (AES Chemunex, bioMérieux France). Some isolates were also identified by MALDI-TOF analysis (MALDI Biotyper, Brucker, France).

The antibiotic susceptibility tests were performed by the disk diffusion method. Susceptibility to 16 antibiotics, including penicillin (aminopenicillin with clavulanic acid), cephalosporins (cefotaxime and ceftazidime), cephamycin (cefoxitin), carbapenems (imipenem and ertapenem), aminoglycosides (kanamycin, tobramycin, gentamycin, streptomycin, and spectinomycin), chloramphenicol, quinolones (nalidixic acid, ofloxacin), tetracycline (doxycycline), and cotrimoxazol was determined for all recovered *E. coli* isolates (CLSI, 2012a). The production of ESBL was assessed by the double-disk synergy test (Jarlier et al., 1988). Isolates were classified as susceptible or resistant to the drug (isolates showing intermediate susceptibility were considered as resistant) according to the Clinical and Laboratory Standards Institute guidelines (CLSI, 2012b).

### Characterization of ESBL-*Ec* by MLST, Detection and Sequencing of *bla* Genes, as Well as Occurrence of Class 1 Integrons (detection of *intl*1) Among These Isolates

DNA of each positive isolate for the production of ESBL was obtained using a rapid extraction method, i.e., boiling 500 μL of a dense culture suspension of each strain and recovering the supernatant after a 5 min centrifugation at 13000 *g*. ESBL-*Ec* were characterized by MLST according the recommendations of Wirth et al. (2006) by amplifying seven house-keeping genes. The PCR fragments were then sequenced according to the Sanger method by Genewiz (Takeley, United Kingdom) and the obtained sequences were analyzed with GeneDoc software (Nicholas and Nicholas, 1997). The alleles and sequence types (ST) were assigned according the *E. coli* MLST website^1^ (Alikhan et al., 2018). All *E. coli* MLST data was deposited at Enterobase.

A phylogenetic tree (Multiple Spanning tree, MS Tree) based on the different ST isolated from water, biofilm and fish gut was obtained with the package available at the Enterobase database (Zhou et al., 2017). This phylogenetic tree was displayed and annotated with the online tool iTOL (Letunic and Bork, 2016). For comparison, ESBL-*Ec* strains isolated from effluents of eight WWTP discharging into the Ouche river, over the same period of time, were included in the tree (Hartmann personal communication, ANSES PNREST CIREC grant 2013-1-254).

qPCR conditions, primers and probes used for the amplification of the *bla_CTX–M_* genes coding CTX-M group 1 and CTX-M group 9 have been previously described (Sabate et al., 2002; Hartmann et al., 2012). The occurrence of class 1 integrons among ESBL-*Ec* strains was estimated by PCR using primers L2 and L3 as previously described (Maguire et al., 2001). Five PCR products were sequenced to confirm the identity with *intl1* sequence. All PCR products were sequenced according to the Sanger method by Genewiz (Takeley, United Kingdom), and compared with those available in the GenBank database using the Blast algorithm^2^. Sequences were deposited in the GenBank database under accession numbers MF926412 through MF926422, MF977504 through MF977517, MF977541 through MF977549 and MG029088, MG029089 for *bla_CTX–M_* genes and MF769720, MF769721, MF769722, MF769723, MF769724 for *intl1* gene.

### Statistical Analyses

Differences in the prevalence of ctxR *Ec* and total *E. coli* between fish species and fish groups were tested using Fisher’s exact test, to compare the proportion of infected individuals between the two sites, or the proportion between the two groups consisting of omnivorous species and predator species, according to their diets as defined by Keith and Allardi (2001). All analyses were performed with JMP software v. 5.01 (SAS Institute Inc., Cary, NC, United States).

## Results

### Distribution of ctxR *Ec* in Environmental Samples (Water and Biofilm) Along the Ouche River

Twelve out of 14 sites were positive for the presence of ctxR *Ec* (yellow dots in Figure 1) in the water sample and nine sites were positive for the biofilm samples (Table 1). Only the two first sampling sites on the Ouche river, located upstream from the first small town, i.e., Bligny-sur-Ouche, didn’t present ctxR *Ec* in both water and biofilm samples. The water contamination with ctxR *Ec* ranged from presence (*i.e*., at least 0.5 bacteria ×100 ml^−1^ water) to 9.5 × 10^2^ CFU 100 ml^−1^ with the maximum observed at site 3# in Bligny-sur-Ouche. The same trend was observed for the biofilm with a range varying from below detection limit (<0.1 bacteria ×100 mg^−1^ biofilm) to 6.6 × 10^2^ CFU 100 mg^−1^ (site 6# in Mâlain). In water samples, the ratio ctxR *E. coli*/total *E. coli* varied from less than 1 to 7%, while in biofilm samples, this ratio varied from less than 1 to 22%.

**Table 1:** Number of CFU (Colony Forming Units) of *E. coli* and ctxR *E. coli* per 100 ml^−1^ water or per 100 mg^−1^ biofilm; when enrichments were done prior to detection, results were expressed as P (Presence) or A (absence) of ctxR *E. coli*.

Site number# location^a^	Water			Biofilm		
	Number of *E. coli*	Number of ctxR *E. coli*	Occurrence of ctxR *E. coli*^b^	Number of *E. coli*	Number of ctxR *E. coli*	Occurrence of ctxR *E. coli*^b^
1# Lusigny-sur-Ouche	0	0	A	16.6	0	A
2# Bligny-sur-Ouche	500	0	A	58.1	0	A
3# Bligny-sur-Ouche	>3640	946.4	NT	>830	498	NT
4# Pont d’Ouche	75	0	P	66.4	8.3	NT
5# Pont de Pany	300	0	P	41.5	0	P
6# Mâlain	>2500	37.5	NT	>2490	664	NT
7# Plombières	12.5	0	P	24.9	0	A
8# Dijon	>1250	0	P	>830	116.2	NT
9# Dijon	>1250	62.5	NT	415	91.3	NT
10# Varanges	175	12.5	NT	149.4	24.9	NT
11# Tart l’Abbaye	250	0	P	41.5	8.3	NT
12# Trouhans	375	12.5	NT	249	8.3	NT
13# Saint-Jean-de-Losne (Saône river)	25	0	P	0	0	A
14# Saint-Jean-de-Losne (harbor)	412.5	0	P	>830	0	A

*^a^Sites are identified by a number and the name of the nearest city (location), ^b^enrichment cultures for ctxR E. coli were analyzed only in case no or very few colonies were observed after direct plating of samples, NT not tested because colonies were obtained without enrichment.*

### Patterns of Abundance and Distribution of ctxR *Ec* in Fish Guts in Two Sites of the Ouche River

One hundred twenty-seven fishes were captured; 20 chubs (*Leuciscus cephalus*), 23 vairones (*Telestes souffia*), 12 loaches (*Barbatula barbatula*), 21 minnows (*Phoxinus phoxinus*), 13 gudgeons (*Gobio gobio*), 23 bullheads (*Cottus gobio*), 6 barbells (*Barbus barbus*), 3 perches (*Perca fluviatilis*), and 6 roaches (*Rutilus rutilus*). The characteristics of fishes sampled for this study are given in Table 2. Since the prevalence of total *E. coli* and ctxR *Ec* in fish guts was low, results were expressed as presence or absence of these bacteria after enrichment of the samples. The prevalence of total *E. coli* and ctxR *Ec* among fish species and from the two sampling sites is presented in Table 3. Grouping of fish species as omnivorous or predator species is given in Table 2.

**Table 2:** Number and size range of fishes sampled in the Ouche river.

	8# Dijon		9# Dijon	
Diet type and fish species	N^a^	Size range (cm)	N^a^	Size range (cm)
**Omnivorous**				
*Leuciscus cephalus*	13	13.7–27.8	7	15.2–24.8
*Telestes souffia*	6	13.2–17.9	17	14.7–19.5
*Phoxinus phoxinus*	10	6.4–7.9	11	6.9–8.3
*Gobio gobio*	9	7.8–11.2	4	8.8–9.5
*Barbus barbus*	6	15.6–26.3	0	
*Rutilus rutilus*	6	11.5–17.6	0	
**Predator**				
*Barbatula barbatula*	9	6.7–8.4	3	6.2–7.5
*Cottus gobio*	9	8.7–12.2	14	9.8–12.3
*Perca fluviatilis*	3	13.4–15.4	0	

*^a^N: number of individuals.*

**Table 3:** Prevalence of total *E. coli* and ctxR *Ec* among fish species in the Ouche river originated from 2 Dijon sites.

	8# Dijon			9# Dijon		
Fish species	N^a^	Number of fishes carrying *E. coli* (%)	Number of fishes carrying ctxR *E. coli* (%)	N^a^	Number of fishes carrying *E. coli* (%)	Number of fishes carrying ctxR *E. coli* (%)
**Omnivorous**						
*Leuciscus cephalus*	13	12 (92.3%)	11 (84.6%)	7	4 (57.1%)	3 (42.8%)
*Telestes souffia*	6	3 (50%)	3 (50%)	17	6 (35.3%)	5 (29.4%)
*Phoxinus phoxinus*	10	3 (30%)	3 (30%)	11	5 (45.5%)	5 (45.5%)
*Gobio gobio*	9	4 (44.4%)	0 (0%)	4	1 (25%)	1 (25%)
*Barbus barbus*	6	4 (66.6%)	3 (50%)	0		
*Rutilus rutilus*	6	3 (50%)	3 (50%)	0		
**Predator**						
*Barbatula barbatula*	9	1 (11.1%)	0 (0%)	3	1 (33.3%)	0 (0%)
*Cottus gobio*	9	0 (0%)	0 (0%)	14	1 (7.1%)	1 (7.1%)
*Perca fluviatilis*	3	0 (0%)	0 (0%)	0		

*^a^N: number of individuals.*

**FIGURE 2:**
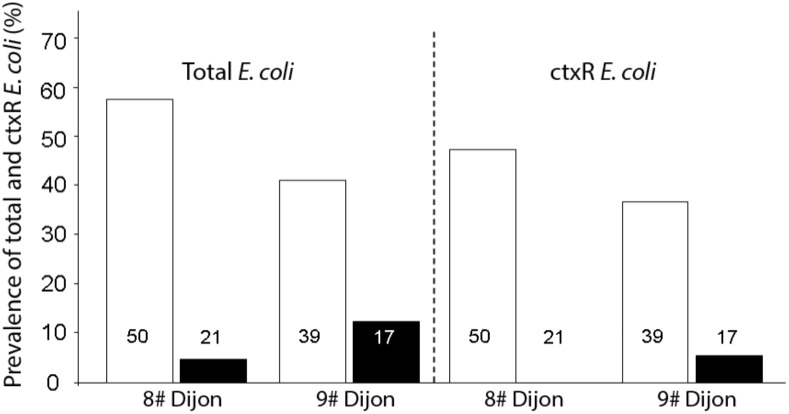
Comparison of the prevalence of total *E. coli* and ctxR *Ec* between omnivorous fish (open bars) and predatory fish (black bars) for the two sites. The number of fish guts studied for each sample is indicated within each bar.

No difference was found in fishes between the two sites concerning the prevalence of total *E. coli* (Fisher’s exact test *p* = 0.5493) and the prevalence of ctxR *Ec* (Fisher’s exact test *p* = 0.5606). However, in the two sites (Figure 2), prevalence of total *E. coli* and ctxR *Ec* between fish species were profoundly variable and seemed strongly influenced by their diet. That is, omnivorous species were significantly more colonized than predator species both for total *E. coli* (Site 8#: Fisher’s exact test *p* < 0.0001, Site 9#: Fisher’s exact test *p* = 0.0001) and ctxR *Ec* (Site 8#: Fisher’s exact test *p* = 0.0351; Site 9#: Fisher’s exact test *p* = 0.0229).

### Phenotyping and Genotyping of ctxR *Ec* Isolated From Fish Guts and Environmental Samples

One hundred and twenty ctxR isolates originated from water, biofilm and fish guts were recovered from TBX plates supplemented with cefotaxime used for enumeration or detection of ctxR *Ec*. Water and biofilm isolates were recovered from the 14 sites and analyzed along the Ouche river, whereas fish isolates originated only from sites eight and nine. Seventy-three ctxR isolates were successfully purified and cryo-conserved. Among those, 52 isolates proved to belong to the *E. coli* species. These 52 isolates were screened for their antibiotic susceptibility and for their ESBL phenotype. Among these, 36 isolates were confirmed to produce ESBL (positive for the double-disk synergy test, and/or because they carried at least one *bla_CTX–M_* gene).

**FIGURE 3:**
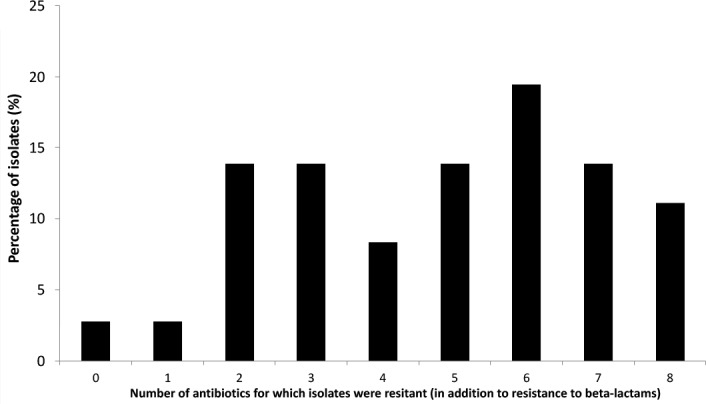
Proportion of ESBL producing *E. coli* isolates harboring additional antibiotic resistances (10 antibiotics tested).

Further analyses were performed only on this subset of 36 ESBL-*Ec* isolates. The antibiotic susceptibility tests revealed that a large proportion of ESBL-*Ec* isolates were a multidrug resistant (MDR) bacteria (Figure 3). Only 3% of the strains were only resistant to third generation cephalosporins (3GC) whereas other isolates harbored additional resistance to between 1 to 8 antibiotics. Eighteen percent (18%) of strains were resistant to six different antibiotics (other than those of the cephalosporin family) and 15% were resistant to eight different antibiotics (Figure 3). MDR ESBL-*Ec* seemed to occur in the various habitats tested (environmental or fish gut). Resistance to different antibiotic families was widespread among isolates: i.e., 92% were resistant to at least one aminoglycoside, 78% were resistant to doxycycline, 56% were resistant to cotrimoxazol, 44% were resistant to at least one quinolone and 28% were resistant to chloramphenicol.

Genotyping of the 36 ESBL-*Ec* isolates that originated from water (14 isolates), biofilm (10 isolates), and fish (12 isolates) along Ouche, were revealed using MLST, and the results are given in Table 4. Among this set of 36 isolates, MLST analysis revealed 22 different sequence types (ST) that can be grouped into seven clonal complexes. Clonal complexes 10 and 23 are the most frequently encountered (respectively 7 and 9 strains out of 36 strains tested) although other clonal complexes are encountered only once. Eleven STs were not affiliated to any clonal complex. Most frequently encountered STs (i.e., ST10 and ST410) were isolated from the three habitats. ST 10 (5554 strains recorded in Enterobase) is a ST encountered worldwide and has frequently been reported in water environments. ST410 (421 strains recorded in Enterobase) is also commonly encountered in water environments. Among these 22 different described STs, 17 STs were already found to occur in water environments (as indicated in Enterodatabase). Noticeably, one isolate originating from a bullhead gut at site 9# in Dijon, belonged to ST 131, which grouped clinical ESBL-*Ec* strains commonly involved in human urinary tract infections and bacteremia worldwide (Table 4).

**Table 4:** Genotyping of ESBL producing *E. coli* originated from the Ouche river.

Strain number^a^	Sampling site^b^	Habitat	Sequence Type (clonal complex when existing)	*bla*_CTX–M_ gene (CTX-M group)	Occurence of *intl1* sequence^c^
MIAE02070	6# Mâlain	Biofilm	10 (10)	15 (1)	−
MIAE02071	8# Dijon	Biofilm	212	1 (1)	+
MIAE02072	9# Dijon	Biofilm	38 (38)	27 (9)	−
MIAE02073	9# Dijon	Biofilm	367 (23)	1 (1)	+
MIAE02074	9# Dijon	Biofilm	540	1 (1)	+
MIAE02075	9# Dijon	Biofilm	1850	1 (1)	+
MIAE02076	9# Dijon	Biofilm	367 (23)	1 (1)	+
MIAE02077	9# Dijon	Biofilm	410 (23)	15 (1)	−
MIAE02078	9# Dijon	Biofilm	2851	15 (1)	+
MIAE02079	9# Dijon	Biofilm	1167	1 (1)	−
MIAE02080	9# Dijon	Biofilm	410 (23)	15 (1)	−
MIAE02081	9# Dijon	Biofilm	10 (10)	1 (1)	+
MIAE02082	10# Varanges	Biofilm	181 (168)	14 (9)	−
MIAE02083	10# Varanges	Biofilm	23 (23)	14 (9)	+
MIAE02084	3# Bligny-sur-Ouche	Water	10 (10)	1 (1)	+
MIAE02085	4# Pont d’Ouche	Water	410 (23)	1 (1)	−
MIAE02086	6# Mâlain	Water	1431	15 (1)	+
MIAE02087	8# Dijon	Water	10 (10)	15 (1)	+
MIAE02088	9# Dijon	Water	1196	1 (1)	−
MIAE02089	11# Tart l’Abbaye	Water	69 (69)	15 (1)	+
MIAE02090	11# Tart l’Abbaye	Water	457	14 (9)	−
MIAE02091	13# Saint-Jean-de-Losne	Water	744	1 (1)	−
MIAE02092	14# Saint-Jean-de-Losne	Water	90 (23)	15 (1)	+
MIAE02093	14# Saint-Jean-de-Losne	Water	90 (23)	15 (1)	+
MIAE02094	8# Dijon	Fish – roach	38 (38)	15 (1)	−
MIAE02095	8# Dijon	Fish – chub	131 (131)	1 (1)	−
MIAE02096	8# Dijon	Fish – chub	617 (10)	15 (1)	+
MIAE02097	8# Dijon	Fish – chub	617 (10)	15 (1)	+
MIAE02098	9# Dijon	Fish – vairone	38 (38)	27 (9)	−
MIAE02099	9# Dijon	Fish – vairone	410 (23)	14 (9)	−
MIAE02100	9# Dijon	Fish – vairone	362	14 (9)	−
MIAE02101	9# Dijon	Fish – vairone	362	14 (9)	−
MIAE02102	9# Dijon	Fish – vairone	540	1 (1)	+
MIAE02103	9# Dijon	Fish – minnow	354 (354)	14 (9)	+
MIAE02104	9# Dijon	Fish –minnow	357	15 (1)	−
MIAE02105	9# Dijon	Fish – chub	10 (10)	15 (1)	+

*^a^Strain numbers are from the MIAE collection hosted at INRA Dijon, UMR Agroecologie. ^b^Site 13# is water from the Saône river at Saint-Jean-de-Losne and site #14 is water from the Saint-Jean-de-Losne harbor. ^c^+ Presence, − absence.*

**FIGURE 4:**
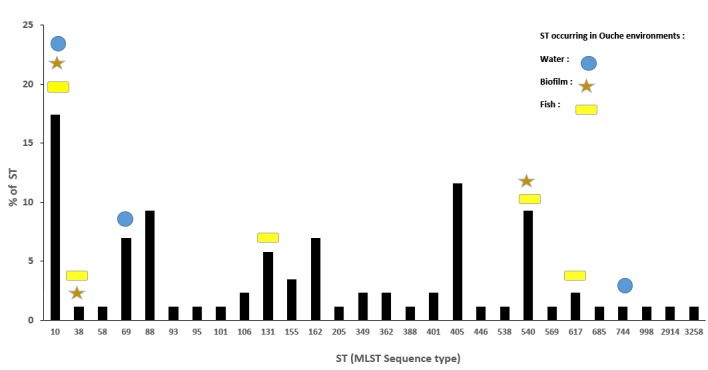
Distributionof ST (%) encountered in the eight WWTP effluents discharging into the Ouche river and their occurrence in environmental samples: water, biofilm, and fish guts.

**FIGURE 5:**
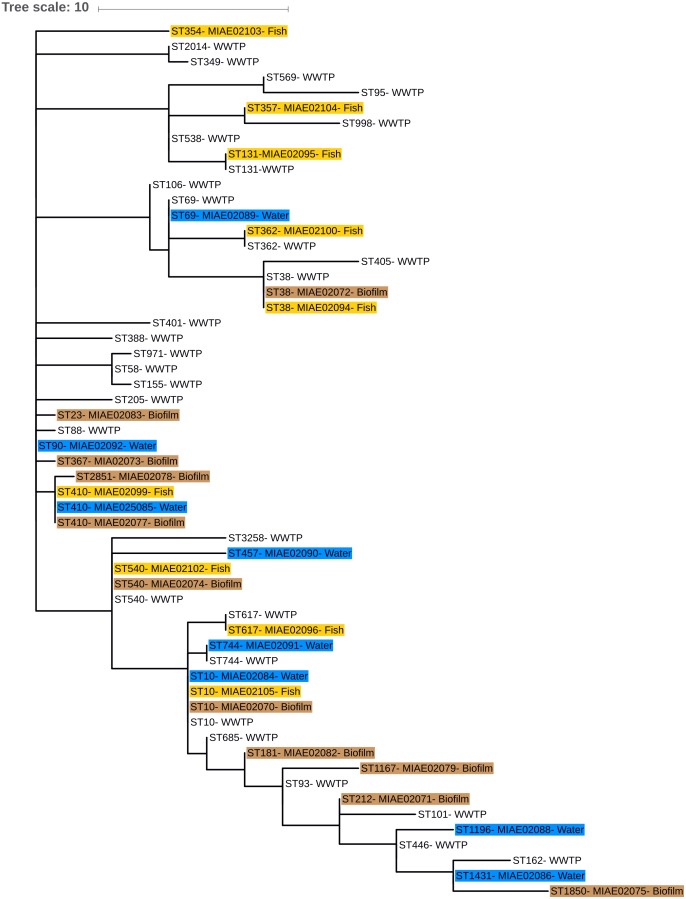
Phylogenetic minimum spanning tree (MS tree) based on MLST sequences of seven housekeeping genes from environmental and WWTP effluents strains. ST numbers from environmental strains are followed by isolate number (see Table 4) and water, biofilm, and fish isolates are highlighted in blue, brown, and yellow, respectively. Only ST number is given for WWTP effluents isolates.

Eighty-six ESBL-*Ec* isolates originating from effluents of the eight WWTP discharging into Ouche river, typed by MLST (Hartmann, personal communication), were compared to environmental strains obtained in this study. WWTP isolates belonged to 28 different STs (Figure 4). Sequence type ST10 was the most frequently encountered ST among WWTP isolates, similar to that observed among the Ouche isolates. ST10 was present in five of the eight WWTP studied. Seven STs occurring in the WWTP effluents were also encountered in the Ouche samples (biofilm, water or fish). However, 14 different STs (including the frequently detected ST410) occurring in the Ouche isolates did not occur in the WWTP effluents. The phylogenetic tree produced from one strain of each ST encountered in the WWTP effluent, water, biofilm or fish gut (Figure 5) reveals a huge diversity in ESBL-*Ec* strains, no ST or clonal complex seemed to be linked with the origin of the sample.

Occurrence of *bla_CTX–M_* genes and the *intl*1 gene in the 36 ESBL-*Ec* isolates were determined by PCR. The *bla_CTX–M_* gene type was determined for each strain by Sanger sequencing (Table 4). Four different *bla*_CTX–M_ genes (bla_CTX–M1,14,15,27_) were observed among the set of 36 isolates. These genes belonged to the two major *bla*_CTX–M_ coding CTX-M group 1 and CTX-M group 9.

No significant difference was found in the proportion of *bla*_CTX–M_ coding CTX-M group 1 and CTX-M group 9 between the isolates coming from water, biofilm and fish guts (χ^2^_2_ = 3.07, *p* = 0.2151). Similarly, among the *bla*_CTX–M_ coding CTX-M group 1, no difference was found in the proportion of *bla*_CTX–M1_ and *bla*_CTX–M15_ between the isolates from water, biofilm and fish (χ^2^_2_ = 2.18, *p* = 0.3360).

Sequences showing 100% identity with the *intl1* sequence occurred in 53% of the strains (Table 4).

## Discussion

The objectives of the study were to monitor the occurrence of total *E. coli* and ESBL-*Ec* in a small watershed (Ouche river) targeting environmental matrices (water, biofilm) as well as fishes that live in the aquatic ecosystem. This watershed is impacted by anthropic activities, i.e., urban wastewater treatment but also by farm effluents that might also impact bacteriological water quality of the river.

### Environmental Dissemination of ctxR *Ec* and Particularly of ESBL-*Ec*

First, water sampling revealed the presence of *E. coli* all along the Ouche river, at all sampling sites excepted from the river source. Interestingly, ctxR *Ec* contaminations of water and biofilm appeared downstream from the first WWTP discharge in the Ouche river (site #3 in Bligny-sur-Ouche, Figure 1). Thus, our observations confirm that WWTP are probably the main source point of water contamination by ctxR *Ec* (Blaak et al., 2014; Brechet et al., 2014). Among ctxR *Ec* isolates, at least 69% produced a CTX-M type ESBL. The other strains (not showing ESBL phenotype) may produce cephalosporinases or other beta-lactamases. The Ouche watershed ecosystem contamination by ESBL-*Ec* seems to be linked to WWTP discharge, which is in agreement with previously published data indicating that discharge of treated wastewater is a source point of ESBL-*Ec* for freshwater ecosystems (Brechet et al., 2014; Czekalski et al., 2014; Vivant et al., 2016). ESBL-*Ec* might thus serve as an indicator of the anthropic impact (urban or farming) in aquatic ecosystems. The ratio of ctxR *Ec*/total *E. coli* seems to be overall higher in biofilm samples than in water samples, thus highlighting the role of biofilm as a potent reservoir for ctxR *Ec*.

### Prevalence of Total *E. coli* and ctxR *Ec* in Fish Guts

Prevalence of total *E. coli* in fish guts varied from 0 to 92% across fish species. Our study revealed that fish diet (predator or omnivore) might significantly influence the prevalence of total *E. coli* as well as ctxR *Ec*. To our knowledge, this is the first description of the occurrence of ESBL-*Ec* in freshwater fishes in France. Our findings agree with previous work showing the occurrence of ESBL-*Ec* in fishes from Swiss lakes (Abgottspon et al., 2014; Zurfluh et al., 2015). Several studies have already shown the presence of ESBL-*Ec* in fish from farmed (Jiang et al., 2012) or wild population in Asia (Hon et al., 2016) and in Africa (Moremi et al., 2016). Although *E. coli* does not seem to be a permanent inhabitant of fish guts (Austin, 2006), these bacteria are able to colonize the fish gut, especially in omnivorous fishes that, at least partially, feed from contaminated biofilms. The higher ratio of ctxR *Ec*/total *E. coli* in biofilm samples compared to water might thus also explain the higher contamination of omnivorous fishes by ctxR Ec and ESBL-*Ec.* Further studies are needed, including time lapse survival of ESBL-*Ec* in the gut of various fish species (omnivorous or predatory), to explore the adaptation of *E. coli* and ESBL-*Ec* to fish gut and potential contamination of fish muscles.

In our survey, the three species of wild predatory fish harbored very little contamination by ESBL-*Ec*. This could be explained by the anatomy and physiology of these species. Indeed, as for most animals, predatory fishes have significantly shorter guts than omnivorous or herbivorous fishes (Keith and Allardi, 2001). This physical characteristic could be less conducive to the survival and persistence of bacteria. Moreover, high gastric acidity of predator fishes could also play a significant role in the lack of persistence of these bacteria. For example, Western (Western, 1971) showed that in C. *gobio* the gastric contents were maintained at pH 2.0 while food remains in the stomach. However, such acidity could not be sufficient to reduce the number of *E. coli.* Indeed, Foster (Foster, 2004) showed that *E. coli* could resist in strongly acidic environments.

Finally, the difference in the number of ESBL-*Ec* between predator and omnivorous fishes may be explained by a combination of two parameters. First, their feeding behavior could be a first filter to contamination, with omnivorous species more susceptible to be contaminated than predatory species, combined with a second filter represented by the difference in the length of their gut, less favorable to the persistence of bacteria in predators.

### Genotyping Analysis

Our study highlighted a noticeable diversity of ESBL-*Ec*, since 22 different STs were detected among the set of 36 environmental and fish isolates analyzed. Some STs (mainly ST10) seem to be ubiquitous and are detected in both environmental and WWTP isolates. However, some STs (namely ST410 and 13 others) occurred only in environmental ESBL-*Ec* and not in those from WWTP effluents. This result might indicate the presence of other sources for ESBL-*Ec*: i.e., farming activities, livestock effluents, manure application in fields. We demonstrated that *bla*_CTX–M_ genes that are dominant in clinical isolates (Sauget et al., 2016), namely *bla*_CTX–M1_, *bla*_*CTX–M*15_ and *bla*_CTXM14,_ also occurred in environmental and fish isolates. No obvious correlation was observed between the ESBL-*Ec* ST and the type of *bla*_CTX–M_ gene carried or with the presence of the *intl1* gene sequence. One fish gut isolate belonged to the well-known ST131 that groups clinical epidemic clones (Sauget et al., 2016) thus indicating that this genotype might occur and/or persist in the environment.

The genetic diversity of ESBL-*Ec* did not seem to corelate with a special habitat or with a sampling site. Whatever the source of our strains, all these STs were already described in a large diversity of habitats and hosts. These STs could group isolates either pathogenically or not for Humans (see text footnote^2^).

Although there was no obvious relationship between the occurrence of the *intl*1 sequence and the multi-resistance phenotype, the potential presence of class 1 integrons in half of the strains might explain the MDR phenotype of some isolates. Further complete sequencing of these integrons might shed light on their potential role in the antibiotic resistance phenotype of environmental strains. Indeed, Gillings et al. (2015) proposed to use the class 1 integron-integrase gene as a potential marker of the anthropic impact on the environment (anthropogenic pollution). Occurrence of clinical class 1 integrons in our strains obviously indicates their human origin and thus reinforces the hypothesis that the main origin of this contamination is due to effluent discharge from WWTP. Such discharges can induce serious public health problems. Indeed, these resistant strains found in freshwater environments might contribute to antibiotic resistance currently spreading in the human population, through the consumption of fish, or by water reuse for crop irrigation, or through recreational activities (bathing). Moreover, aquatic environments might act as long term or permanent reservoirs for antibiotic resistant bacteria and/or antibiotic resistance genes. Indeed, in their review, Marti et al. (2014) pointed out that bacteria found in natural aquatic ecosystems are organized in biofilms, which facilitate their survival, dispersal, but also, due to high cell density and close proximity, the possible horizontal gene transfer (HGT) between bacteria. Thus, biofilms can serve as long-term reservoirs for antibiotic resistant genes and contribute to the emergence and the dissemination of multi resistant strains.

In this scheme, fishes, and especially fishes that feed on biofilm, could host strains and might be sentinels of environmental contamination through antibiotic resistant bacteria. However, further studies are needed to evaluate the duration of survival of ESBL-*Ec* in the environment and in fish guts. Finally, the fitness of ESBL-*Ec* versus wild type *E. coli* under environmental and fish gut conditions should be analyzed.

At the same time, this study emphasizes the need for complementary treatments of WWTP effluents (tertiary treatments). These treatments should be implemented in order to decrease the spread of such antibiotic resistant isolates. Various tertiary treatments might be implemented: i.e., constructed wetlands [combination of open water ponds and planted bed filters as described by Vivant et al. (2016)], UV treatment, effluent filtration or ozonation.

## Ethics Statement

Electrofishing was performed in accordance with the recommendations of the French National Agency for Water and Aquatic Environments (ONEMA) and authorized by the prefecture from Côte d’Or (France). This study conforms to the legal requirements of France. The experiment has received the agreement of the Animal Care and Ethical Committee of the Université de Bourgogne, Dijon, France.

## Author Contributions

LB had substantial contributions to the conception or design of the work as well as for the drafting of the manuscript. EB had done the practical work and analyzed the results. GD had substantial contributions to the acquisition, analysis, and interpretation of data for the work and also for the drafting of the manuscript. SM contributed to the acquisition of data. CN revised the manuscript critically for important intellectual content. JM provided approval for publication of the content. AH had substantial contributions to the conception or design of the work and to the drafting of the manuscript.

## Conflict of Interest Statement

The authors declare that the research was conducted in the absence of any commercial or financial relationships that could be construed as a potential conflict of interest.

## Footnotes

1 http://enterobase.warwick.ac.uk/species/index/ecoli

2 http://blast.ncbi.nlm.nih.gov/Blast.cgi
